# A Novel Attitude Determination System Aided by Polarization Sensor

**DOI:** 10.3390/s18010158

**Published:** 2018-01-09

**Authors:** Wei Zhi, Jinkui Chu, Jinshan Li, Yinlong Wang

**Affiliations:** Key Laboratory for Micro/Nano Technology and System of Liaoning Province, Dalian University of Technology, Dalian 116024, China; zhiwei_1983@mail.dlut.edu.cn (W.Z.); jinshanl@mail.dlut.edu.cn (J.L.); wangyinlong@mail.dlut.edu.cn (Y.W.)

**Keywords:** polarization sensor, quad-rotor UAV, E-vector direction, solar vector

## Abstract

This paper aims to develop a novel attitude determination system aided by polarization sensor. An improved heading angle function is derived using the perpendicular relationship between directions of E-vector of linearly polarized light and solar vector in the atmospheric polarization distribution model. The Extended Kalman filter (EKF) with quaternion differential equation as a dynamic model is applied to fuse the data from sensors. The covariance functions of filter process and measurement noises are deduced in detail. The indoor and outdoor tests are conducted to verify the validity and feasibility of proposed attitude determination system. The test results showed that polarization sensor is not affected by magnetic field, thus the proposed system can work properly in environments containing the magnetic interference. The results also showed that proposed system has higher measurement accuracy than common attitude determination system and can provide precise parameters for Unmanned Aerial Vehicle (UAV) flight control. The main contribution of this paper is implementation of the EKF for incorporating the self-developed polarization sensor into the conventional attitude determination system. The real-world experiment with the quad-rotor proved that proposed system can work in a magnetic interference environment and provide sufficient accuracy in attitude determination for autonomous navigation of vehicle.

## 1. Introduction

As an input parameter of the position control loop in navigation control systems, the attitude information is an important parameter for aircraft autonomous navigation. The inertial navigation equipment with the characteristic of good covert and autonomous function is widely used in navigation systems. However, application of high precision inertial navigation systems (INSs) in small carriers and low-cost aircrafts has been significantly hampered because of their large size and high cost. With the development of Micro-Electro-Mechanical Systems (MEMSs), the MEMS inertial sensors with the advantages of small size and low cost have been widely used in small aircrafts. However, due to the disadvantage of low measuring precision, a single MEMS inertial sensor is not sufficient to achieve satisfactory precision. On the other hand, high accuracy attitude angles can be obtained by MEMS gyroscope which performs correction using the geomagnetic and gravity vectors and where the conventional attitude reference system has the same combination mode [1,2,3,4,5,6]. Although the gravity vector can effectively modify the attitude angle information [7], the geomagnetic vector used to correct the heading angle cannot accurately point to the geographical North pole because of magnetic declination. The International Geomagnetic Reference Field (IGRF) model can be used to calculate magnetic declination; nevertheless, the accuracy of joint local geomagnetic field model and geomagnetic map still could not meet the requirements for high precision navigation. Furthermore, in the case of large latitudes, due to the Earth magnetic field, there will be a large error in the output of the geomagnetic sensor.

In recent years, more and more scholars have paid attention to the method for correction of heading information using the light vector with polarization characteristics [8]. Similar to the navigation based on the Earth magnetic field, this method also uses a unique property of the Earth, the stable polarization pattern model, which arises due to scattering of sunlight in the atmosphere. Unlike the Earth magnetic field, the polarization pattern is relatively stable at any position on the Earth [9,10]. The method of using the polarization mode to determine a heading angle also has the advantage that error does not accumulate with time. Recently, many scholars have studied the application of polarization pattern model in the mobile carrier. Referring to the special structure used for measuring the polarization in a compound eye of insects. Lambrino’s team used the photovoltaic components to produce a set of polarization-opponent units [11], which were applied to the ground robot and the mechanism of biological sensing polarization information for robot navigation was verified. Chu’s team applied the polarization sensor to the autonomous navigation of outdoor robot [12]. The experimental results showed that polarized light can be used as a reference for navigation system and proved that angle information from polarization sensor has no cumulative error. Hu’s team proposed a new algorithm for vehicle navigation by combining polarization sensor with stereo camera and inertial devices and results showed that proposed algorithm has high precision [13]. The polarization sensor was applied to the navigation of the Unmanned Aerial Vehicle (UAV) in [14] and experimental results showed that polarization sensor can effectively measure vehicle heading angle when UAV is horizontal. However, the existing studies on algorithms for obtaining the heading information from polarization sensor are based on the assumption that sensor is installed horizontally. The benefit of such a principle is that calculation process of heading angle is greatly simplified. However, a tilt action is unavoidable when vehicles make hill-climbing or turn. In that case, the polarization sensor mounted on a vehicle cannot stay in horizontal position, namely and if a simplified algorithm is used there will be a lot of error. To solve these problems, an improved method for heading angle calculation based on the perpendicular relation between direction of the solar vector and direction of the vector of maximum polarized light, is presented in this paper. Using the attitude angle provided by MEMS accelerometer, the output model of polarization sensor can be constructed in any posture. In order to achieve high accuracy, the Extended Kalman filter (EKF) based on the attitude quaternion is introduced to fuse the information from polarization sensor, MEMS accelerometer and gyroscope.

The main contributions of this paper are as follows. Firstly, an improved heading angle function is derived using the perpendicular relationship between direction of E-vector of linearly polarized light and direction of solar vector in the atmospheric polarization distribution model. Secondly, the EKF with quaternion differential equation as a dynamic model is applied to fuse the data from sensors and the covariance functions of filter process and measurement noises are deduced in detail. Thirdly, to verify the effectiveness of implemented algorithm, the proposed attitude determination system was subjected to two tests, namely the indoor strong magnetic interference test and outdoor flight test near the high-tension wires. 

The rest of paper is organized as follows. Section 2 illustrates the block diagram of proposed attitude determination system and provides a review of coordinate frames and notations applied in this study. Section 3 discusses the measurement models of polarization sensor and accelerometer modules. Section 4 introduces the attitude estimation algorithm based on EKF. Section 5 presents experimental results. Section 6 concludes the paper and gives the guidelines for our future work.

## 2. System Framework and Coordinates

### 2.1. System Framework

**Figure 1:**
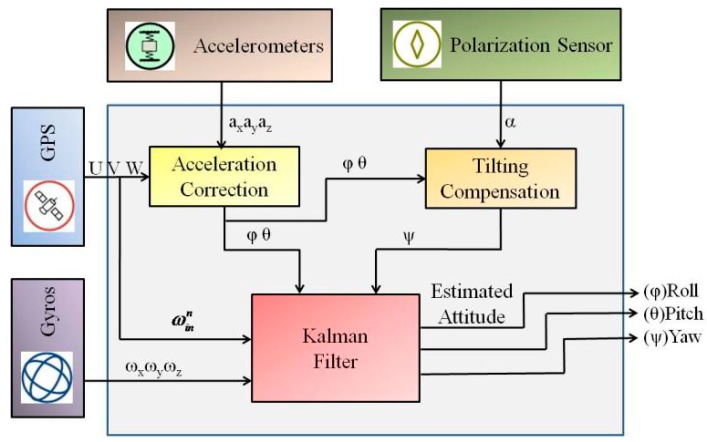
The schematic diagram of proposed algorithm.

The schematic diagram of algorithm proposed in this paper is shown in Figure 1. The novel attitude determination system includes the low-cost MEMS inertial measurement unit (MIMU), polarization sensor and GPS receiver. The EKF is adopted to integrate measurement results. The quaternion differential equation is a dynamic EKF model with four parameters as the state variables. The roll angle (*φ*) and pitch angle (*θ*) are calculated based on gravitational acceleration derived from accelerometer signals after motion acceleration compensation; the heading angle (*ψ*) is determined from the polarization sensor data; and these three angels are selected as the measurement of EKF. The velocity information (*W*, *U*, *V*) obtained from the GPS data is used to correct the output of MIMU to achieve the gravity force decomposition (*g_x_*, *g_y_*, *g_z_*) and rotation rate ($\omega_{in}^{n}$) of the navigation frame with respect to the inertial frame. The heading angle is acquired by polarization sensor with the aid of correction from accelerometers. All devices used for this novel attitude determination are small in size and have low power consumption, which is suitable for small air vehicles.

### 2.2. Coordinate Systems and Notations

There are three separate reference frames in this paper: navigation frame, body frame and polarization sensor frame.

The navigation frame (n-frame) represents a local geographic frame with the origin at the navigation system and axes aligned with the directions of north and east and local vertical direction (down).

The body frame (b-frame) represents an orthogonal frame rigidly attached to the vehicle with the origin co-located with the n-frame and axes aligned with the roll, pitch and yaw axes of the vehicle. The inertial sensors are fixed to the b-frame.

The polarization sensor frame (s-frame) is a right-handed frame that coincides with the b-frame of vehicle. 

**Figure 2:**
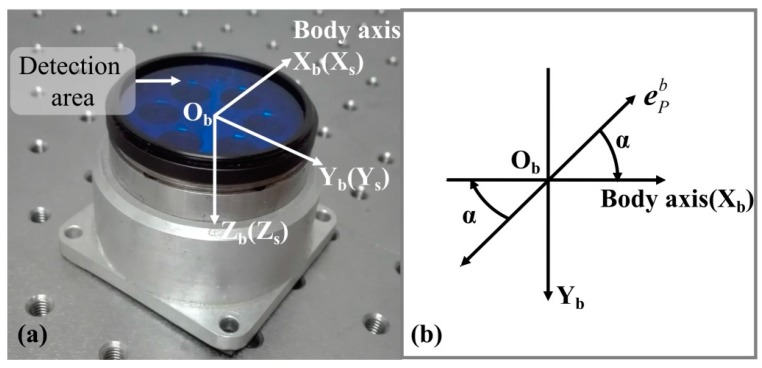
The polarization sensor and definition of sensor output.

The polarization sensor used in this study is shown in Figure 2a. The detection area of polarization sensor corresponds to the X_b_-Y_b_ plane of b-frame. There is a reference direction in the detection area aligning with the positive x-axis of the b-frame, as shown in Figure 2. The angle α is the output of the polarization sensor, which represents the clockwise angle between the reference direction of sensor and E-vector direction of the incident light, as shown in Figure 2b. It should be noted that E-vector direction is a special vector, which has double direction, so the angle α could also represents its opposite angle. 

## 3. Sensor Modeling

### 3.1. The Improved Model of Polarization Sensor

**Figure 3:**
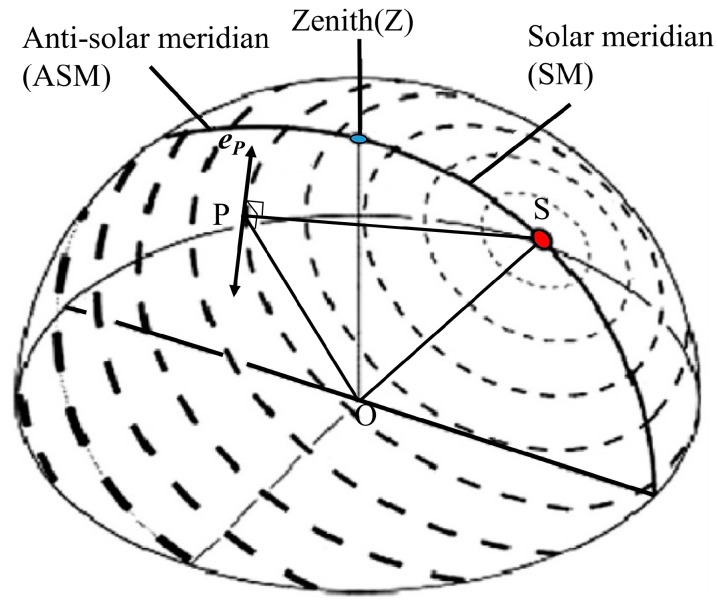
The 3D pattern of polarization in the sky. Orientation and width of the bars depict direction and degree of polarization. A remarkable pattern characteristic is that there is a symmetry line going through the Sun (S) and zenith (Z). On the side of the Sun it is called the solar meridian (SM) and on the opposite side it is called the anti-solar meridian (ASM) [11]. The point O denotes the position of the observer. The point P represents any position observed in the sky. The vector **e**_P_ represents the maximum polarization direction of point P, which is a special vector with double direction.

During the sunlight transmission through the atmosphere, the polarized light is generated by atmospheric scattering of air molecules and aerosol particles. The polarized light has various polarization states in the sky which form the skylight polarization distribution pattern shown in Figure 3. The distribution pattern has the following two characteristics. Firstly, it has two symmetrical axes, the maximum polarization axis which is at 90-angle-distance from the Sun and the solar meridian axis that goes through the Sun (S) and zenith (Z). Secondly, the E-vector direction on the solar meridian is perpendicular to the solar meridian. A relatively stable skylight polarization distribution pattern exists in the sky at a certain position during a specific time period, even in the dawn and dusk [15]. In the literature, for describing the polarization distribution pattern, the vibration direction of the polarized light represents an important parameter, which is also named the polarization direction vector. The maximum polarization direction vector (also named as the E-vector direction) has a characteristic which is in accordance with the principle of Rayleigh scattering, that E-vector direction is always perpendicular to the scattering plane [16] determined by observer (O), position observed in the sky (P) and Sun (S), Figure 3. Therefore, the vehicle heading angle can be obtained by identifying the geometric relationship between the reference direction of polarization sensor and E-vector direction in the sky.

So far, scientists have made a lot of theoretical and experimental investigations on skylight polarization and obtained results have been used in autonomous orientation and navigation. Most of the algorithms in the literature represent simplified calculation on heading angle [17,18,19]; however, these methods have certain limitations, namely they are applicable only to the horizontally placed polarization sensors. These algorithms can acquire high-accuracy heading angle but the aircraft must keep horizontal state all the time which is impossible in practice. Consequently, these algorithms can have a great error. In the following section, an improved heading calculation method with the aid of correction by accelerometers, which is applicable to the polarization sensors at any attitude, is proposed.

**Figure 4:**
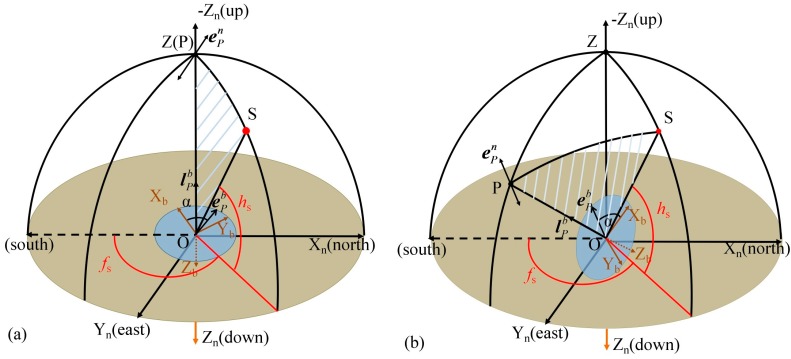
Description of E-vector direction for different attitudes of polarization sensor: (**a**) Polarization sensor is placed horizontally; (**b**) Polarization sensor is on a tilt. S denotes the position of the Sun; O is the position of the observer; P represents the observed point in the sky; Z is the zenith, f_s_ is the solar azimuth and h_s_ is the solar altitude angle. The vector **e**_P_ represents the E-vector direction of the observed position. The detection area of polarization sensor is in X_b_-O-Y_b_ plane.

As mentioned previously, according to the principle of Rayleigh scattering, the E-vector direction is always perpendicular to the scattering plane [16], determined by the observer (O), observed point in the sky (P) and Sun (S), the shaded area in Figure 4. The polarization sensor is placed at the observer’s position and its center overlaps with the point O. During the sunlight transmission through the atmosphere, the polarized light is generated by atmospheric scattering of air molecules and aerosol particles at the point P. The polarized light is detected by polarization sensor.

When the polarization sensor is placed horizontally, the detection area of sensor is focused to the zenith, as shown in Figure 4a. The E-vector direction of incident light is perpendicular to the solar meridian because the solar meridian is in the scattering plane. The angle between the reference direction of polarization sensor and solar meridian is either α + π/2 or α − π/2. Since solar meridian can be expressed by solar altitude (*h*_s_) and solar azimuth (*f*_s_), which could be calculated in theory, the heading angle of the reference direction with respect to the Earth south would be *f*_s_ + α + π/2 or *f*_s_ + α − π/2. This simplified model has been adopted in many studies. 

If sensor is tilted, as shown in Figure 4b, the E-vector direction will not be always perpendicular to the solar meridian and the heading angle could not be acquired by means of simple arithmetic. Therefore, an improved heading angle algorithm should be discussed. As described previously, the solar vector is always in scattering plane, so the E-vector direction is perpendicular to the solar vector. In navigation frame, their relationship could be represented by:

(1) $$e_{P}^{n}•a_{OS}^{n}=0$$

where $a_{OS}^{n}$ represents the solar vector represented in navigation frame and $e_{P}^{n}$ represents the E-vector direction represented in navigation-frame (n-frame) defined as:

(2) $$e_{P}^{n}={\left[\begin{array}{ccc} e_{Px}^{n} & e_{Py}^{n} & e_{Pz}^{n} \end{array}\right]}^{T}$$

As it can be seen in Figure 4, the viewing direction of sensor **l**_P_ is always in scattering plane, thus, the E-vector direction is perpendicular to **l**_P_. Since the detection area of polarization sensor always focuses to the observed point P, **l**_P_ is perpendicular to X_b_-O-Y_b_ plane. The E-vector direction of point *P* is always parallel to X_b_-O-Y_b_ plane, so the E-vector direction represented in b-frame can be defined as:

(3) $$e_{P}^{b}={\left[\begin{array}{ccc} \cos\alpha & \sin\alpha & 0 \end{array}\right]}^{T}$$

Then, the E-vector direction represented in n-frame is:

(4) $$e_{P}^{n}{=C}_{b}^{n}e_{P}^{b}$$

where $C_{b}^{n}$ is the DCM from b-frame to n-frame.

In astronomy, there are clear definitions of solar azimuth and solar altitude angles which are respectively denoted by *f_s_* and *h_s_*. The calculation of solar azimuth and solar altitude angles can be found in [20]. The solar vector $a_{OS}^{n}$ expressed in n-frame can be calculated by solar azimuth and solar altitude angles and the corresponding formula is:

(5) $$a_{OS}^{n}=\left[\begin{array}{c} a_{OS}^{n}{}_{x}\\ a_{OS}^{n}{}_{y}\\ a_{OS}^{n}{}_{z} \end{array}\right]=\left[\begin{array}{c} \cos(h_{s})\cos(f_{s})\\ \cos(h_{s})\sin(f_{s})\\ \sin(h_{s}) \end{array}\right]$$

Combining Equations (1) and (4) with Equation (5), the improved heading angle *ψ* can be determined by:

(6) ψ=arcsinb+arctanc

where:

$$b=\frac{\cos\alpha \sin\theta -\sin\alpha \cos\theta \sin\varphi }{\mathrm{ctan}h_{s}\sqrt{{\left(\sin\alpha \sin\varphi \sin\theta +\cos\alpha \cos\theta \right)}^{2}+\cos^{2}\varphi \sin^{2}\alpha }}$$

$$c=\frac{\cos\alpha \cos\theta \cos f_{s}+\sin\alpha \sin\varphi \sin\theta \cos f_{s}+\cos\varphi \sin\alpha \sin f_{s}}{\cos\alpha \cos\theta \sin f_{s}+\sin\alpha \sin\varphi \sin\theta \sin f_{s}-\cos\varphi \sin\alpha \cos f_{s}}$$

According to the above equations, for any combination of pitch and roll angles, the heading angle of vehicle can be calculated. It should be noted that if sensor is placed horizontally (*θ* = 0, *φ* = 0), the function *b* in Equation (6) will be equal to zero. Then, Equation (6) can be rewritten by:

(7) ψ=arctanc

Simplifying Equations (7), the result is $\psi_{1}=\pi /2-\alpha -f_{s}$ or $\psi_{2}=-\pi /2-\alpha -f_{s}$, which is in accordance with aforementioned values.

As already mentioned, E-vector direction is a special vector with double direction, consequently, there are two solutions of Equation (7). However, this method cannot be used in practical engineering directly. Therefore, before using these data, a simple decision is needed. In this paper, gyroscope data are used as a reference. Although there are accumulated errors in gyroscope results, the accuracy after correction is very high and the difference between two solutions of Equation (7) is large, thus, this method is feasible.

### 3.2. The Attitude Determination Based on Theory of Gravity

The roll and pitch angles of vehicle can be found from vehicle’s gravity vector obtained by accelerometer. Knowing the gravitational force components expressed in b-frame, denoted as *g_x_*, *g_y_* and *g_z_*, the attitude angles *φ* and *θ* [21] can be calculated by:

(8) $$\theta =\tan^{-1}\left(\frac{-g_{x}\cos\phi }{g_{z}}\right)$$

However, *g_x_*, *g_y_* and *g_z_* cannot be measured directly. The accelerometer output is the specific force which include gravity acceleration and motion acceleration that cannot be distinguish by the accelerometer itself. Thus, GPS is used to measure velocity needed to compute the motion accelerations [22]. As a result, the gravitational acceleration can be determined by subtracting the motion accelerations from the specific force of accelerometer. The gravitational force components [21] are defined by:

(9) $$\begin{array}{l} g_{x}=a_{x}-\dot{U}-\left(W\omega_{y}-V\omega_{z}\right),\\ g_{y}=a_{y}-\dot{V}+\left(U\omega_{z}-W\omega_{x}\right),\\ g_{z}=a_{z}-\dot{W}+\left(V\omega_{x}-U\omega_{y}\right), \end{array}$$

where (*U*, *V*, *W*) are the motion velocities and ($\omega_{x}$, $\omega_{y}$, $\omega_{z}$) are the pitch, roll and yaw rates, respectively. The method of deriving (*U*, *V*, *W*) from GPS appears in a lot of literatures, therefore it is not described in this article.

## 4. The Data Fusion Algorithm Based on EKF 

### 4.1. The Kinematical Model Using Quaternion

The relative rotation rate of vehicle to the navigation frame $\omega_{nb}^{b}={\left[\begin{array}{ccc} \omega_{x} & \omega_{y} & \omega_{z} \end{array}\right]}^{T}$ can be calculated by [23]:

(10) $$\omega_{nb}^{b}=\omega_{ib}^{b}-C_{n}^{b}\omega_{in}^{n}$$

where, $\omega_{ib}^{b}$ is the rotation rate of the body with respect to the inertial frame, which is measured by gyroscopes and $\omega_{in}^{n}$ is the rotation rate of the navigation frame with respect to the inertial frame, which is computed by:

(11) $$\omega_{in}^{n}=\left(\begin{array}{c} \Omega \cos L+\frac{v_{E}}{R_{0}+h}\\ -\frac{v_{N}}{R_{0}+h}\\ -\Omega \sin L-\frac{v_{E}\tan L}{R_{0}+h} \end{array}\right)$$

where $v_{N}$ and $v_{E}$ are the north and east components of vehicle velocity with respect to the Earth, *L* is the vehicle latitude, *h* is the vehicle height from the ground, *R*_0_ is the mean radius of the Earth and Ω is the rotation rate of the Earth.

The quaternion method is universally applied to calculation of attitude angle [24] and it uses a quaternion **q** = [*q*_0_ *q*_1_ *q*_2_ *q*_3_]^T^ to determine the aircraft attitude. The relationship between the direction cosine matrix and quaternion is given by:

(12) $$C_{b}^{n}=\left[\begin{array}{ccc} q_{0}^{2}+q_{1}^{2}-q_{2}^{2}-q_{3}^{2} & 2\left(q_{1}q_{2}-q_{0}q_{3}\right) & 2\left(q_{1}q_{3}+q_{0}q_{2}\right)\\ 2\left(q_{1}q_{2}+q_{0}q_{3}\right) & q_{0}^{2}-q_{1}^{2}+q_{2}^{2}-q_{3}^{2} & 2\left(q_{2}q_{3}-q_{0}q_{1}\right)\\ 2\left(q_{1}q_{3}-q_{0}q_{2}\right) & 2\left(q_{2}q_{3}+q_{0}q_{1}\right) & q_{0}^{2}-q_{1}^{2}-q_{2}^{2}+q_{3}^{2} \end{array}\right]$$

The four parameters of quaternion follow the constraint equation; thus, it can be written that:

(13) $$q_{0}^{2}+q_{1}^{2}+q_{2}^{2}+q_{3}^{2}=1$$

The kinematical equation of quaternion regarding the vehicle rate rotation with respect to the navigation frame can be described by:

(14) $$\dot{q}=\frac{1}{2}\Omega q,$$

where:

$$\Omega =\left[\begin{array}{cccc} 0 & -\omega_{x} & -\omega_{y} & -\omega_{z}\\ \omega_{x} & 0 & \omega_{z} & -\omega_{y}\\ \omega_{y} & -\omega_{z} & 0 & \omega_{x}\\ -\omega_{z} & -\omega_{y} & -\omega_{x} & 0 \end{array}\right],$$

and ($\omega_{x}$, $\omega_{y}$, $\omega_{z}$) is the set of rates of angular, motion.

The relationship between attitude angles and quaternion can be presented by:

(15) $$\left[\begin{array}{c} \phi \\ \theta \\ \psi \end{array}\right]=\left[\begin{array}{c} \tan^{-1}\left(\frac{2(q_{0}q_{1}+q_{0}q_{1})}{q_{0}^{2}-q_{1}^{2}-q_{2}^{2}+q_{3}^{2}}\right)\\ \sin^{-1}2(q_{0}q_{3}-q_{1}q_{2})\\ \tan^{-1}\left(\frac{2(q_{0}q_{3}+q_{1}q_{2})}{q_{0}^{2}+q_{1}^{2}-q_{2}^{2}+q_{3}^{2}}\right) \end{array}\right]$$

Therefore, the attitude angles can be obtained by solving the kinematical equation of quaternion.

### 4.2. State Model and Observation Model of Kalman Filter

The Kalman filter is applied in the proposed attitude system for data fusion. The quaternion kinematical Equation (14) regarding the vehicle rotation rate is chosen as a state equation of Kalman filter. The Equation (15) which describes the relationship between attitude angles and quaternion is selected as a measurement equation of Kalman filter [21]. Thus, the Kalman filter can be represented by:

(16) $$\begin{array}{l} \dot{X}=AX+W\left(t\right),\\ Z=h(X)+V(t). \end{array}$$

where ***X*** = [*q*_0_ *q*_1_ *q*_2_ *q*_3_]^T^ is the system state vector, ***Z*** = [*φ θ ψ* 1] is the measurement vector and ***W***(*t*) and ***V***(*t*) are the process and measurement noises, respectively. In this paper, the gyroscope biases are not estimated because of the necessity to keep the state vector size at minimum for a lower computational load. Lower computational load is a requirement regarding the limits of the microprocessor. The state transition matrix *A* in the state equation is defined as:

(17) $$A=\left[\begin{array}{cccc} 0 & \omega_{z} & -\omega_{y} & \omega_{x}\\ -\omega_{z} & 0 & \omega_{x} & \omega_{y}\\ \omega_{y} & -\omega_{x} & 0 & \omega_{z}\\ -\omega_{x} & -\omega_{y} & -\omega_{z} & 0 \end{array}\right]$$

And the nonlinear function *h*(*X*) in the measurement equation is defined as:

(18) $$h(X)=\left[\begin{array}{c} \tan^{-1}\left(\frac{2(q_{0}q_{1}+q_{0}q_{1})}{q_{0}^{2}-q_{1}^{2}-q_{2}^{2}+q_{3}^{2}}\right)\\ \sin^{-1}2(q_{0}q_{3}-q_{1}q_{2})\\ \tan^{-1}\left(\frac{2(q_{0}q_{3}+q_{1}q_{2})}{q_{0}^{2}+q_{1}^{2}-q_{2}^{2}+q_{3}^{2}}\right)\\ q_{0}^{2}+q_{1}^{2}+q_{2}^{2}+q_{3}^{2} \end{array}\right]$$

where the last row is added to satisfy the quaternion norm constraint, which originates from Equation (13).

In the measurement vector *Z*, the pitch angle *θ* and roll angle *φ* are calculated by the gravitational acceleration components defined by Equation (8) and the heading angle *ψ* is determined by Equation (6).

In real time computation, the Euler’s method is used to discretize the dynamical system Equation (16) and the discretion equation can be expressed by:

(19) $$\begin{array}{l} X_{k+1}=F_{k+1}X_{k}+W_{k},\\ Z_{k+1}=h(X_{k+1})+V_{k}. \end{array}$$

where *T* is the sampling period and *F_k__+_*_1_ is defined by the following form [25]:

(20) $$F_{k+1}=\exp(\frac{1}{2}\Omega_{k}T)$$

(21) $$\Omega_{k}=\left[\begin{array}{cccc} 0 & \omega_{z} & -\omega_{y} & \omega_{x}\\ -\omega_{z} & 0 & \omega_{x} & \omega_{y}\\ \omega_{y} & -\omega_{x} & 0 & \omega_{z}\\ -\omega_{x} & -\omega_{y} & -\omega_{z} & 0 \end{array}\right]$$

Since measurement function *h*(*X_k__+_*_1_) is nonlinear, the Extended Kalman filter is introduced to deal with the nonlinear filter. After initialization, the linearized measurement matrix *H_k__+_*_1_ is acquired by appropriately truncating Taylor series expansion based on the system state vector and the matrix *H_k__+_*_1_ can be written as [21]:

(22) $$H_{k+1}={\frac{\partial h}{\partial x}|}_{{\hat{x}}^{-}}=\left[\begin{array}{cccc} \frac{\partial \phi }{\partial x_{0}} & \frac{\partial \phi }{\partial x_{1}} & \frac{\partial \phi }{\partial x_{2}} & \frac{\partial \phi }{\partial x_{3}}\\ \frac{\partial \theta }{\partial x_{0}} & \frac{\partial \theta }{\partial x_{1}} & \frac{\partial \theta }{\partial x_{2}} & \frac{\partial \theta }{\partial x_{3}}\\ \frac{\partial \psi }{\partial x_{0}} & \frac{\partial \psi }{\partial x_{1}} & \frac{\partial \psi }{\partial x_{2}} & \frac{\partial \psi }{\partial x_{3}}\\ x_{0} & x_{1} & x_{2} & x_{3} \end{array}\right]$$

In the iterative processing, the state transition matrix *F_k__+_*_1_ and system process noise covariance are calculated and then the predictive state and predictive covariance matrix are obtained. Afterwards, the gain matrix and optimal estimation are determined. In order to carry out the next loop, the covariance matrix of an optimal estimation should be also calculated. In the following section, the variances of both measurement noises and process noises are described in detail.

### 4.3. Noise Parameters Determination

During the Kalman filter design, in order to get the smallest estimation error variance, the covariances of process and measurement noises are applied to parameters design. Thus, the accuracy requirement for noise variance must be very high. 

In this paper, to consider the attitude determination, we assume that process noises basically originate from gyroscope outputs ($\omega_{x}$, $\omega_{y}$, $\omega_{z}$). In actually, the rotation rates mentioned before this section are all ideal values and are unknown. In this section, they will be represented by (${\bar{\omega }}_{x}$, ${\bar{\omega }}_{y}$, ${\bar{\omega }}_{z}$). Therefore, it is assumed that $\omega_{x}={\bar{\omega }}_{x}+{\tilde{\omega }}_{x}$, $\omega_{y}={\bar{\omega }}_{y}+{\tilde{\omega }}_{y}$ and $\omega_{z}={\bar{\omega }}_{z}+{\tilde{\omega }}_{z}$ and the Equation (20) can be rewritten [25] as:

(23) $$X_{k+1}={\bar{F}}_{k+1}X_{k}$$

(24) $${\bar{F}}_{k+1}=\exp(\frac{1}{2}{\bar{\Omega }}_{k}T)=\exp(\frac{1}{2}\Omega_{k}T-\frac{1}{2}{\tilde{\Omega }}_{k}T)$$

(25) $${\tilde{\Omega }}_{k}=\left[\begin{array}{cccc} 0 & {\tilde{\omega }}_{z} & -{\tilde{\omega }}_{y} & {\tilde{\omega }}_{x}\\ -{\tilde{\omega }}_{z} & 0 & {\tilde{\omega }}_{x} & {\tilde{\omega }}_{y}\\ {\tilde{\omega }}_{y} & -{\tilde{\omega }}_{x} & 0 & \omega_{z}\\ -{\tilde{\omega }}_{x} & -{\tilde{\omega }}_{y} & -{\tilde{\omega }}_{z} & 0 \end{array}\right]$$

where ${\tilde{\omega }}_{x}$, ${\tilde{\omega }}_{y}$ and ${\tilde{\omega }}_{z}$ are the deviations of $\omega_{x}$, $\omega_{y}$ and $\omega_{z}$, respectively.

Then, the Equation (24) is developed by the formula of Taylor series and higher order terms are neglected, thus it can be represented by:

(26) $${\bar{F}}_{k+1}=\exp(\frac{1}{2}\Omega_{k}T)(1-\frac{1}{2}{\tilde{\Omega }}_{k}T)$$

Therefore, the Equation (25) can be rewritten as:

(27) $$X_{k+1}=F_{k+1}X_{k}+W_{k}$$

and,

(28) $$W_{k}=-\frac{1}{2}F_{k+1}\tilde{\Omega }X_{k}=-\frac{1}{2}F_{k+1}\left[X_{k}⊗\right]\tilde{\omega }$$

where, $\tilde{\omega }=\left[\begin{array}{ccc} {\tilde{\omega }}_{x} & {\tilde{\omega }}_{y} & {\tilde{\omega }}_{z} \end{array}\right]$ is the error vector of the angular velocity and $\left[X_{k}⊗\right]$ is the anti-symmetric matrix of the quaternion.

Hence, the process noise variance *Q_k_* can be obtained by the following form:

(29) $$Q_{k}=E[\tilde{\omega }{\tilde{\omega }}^{T}]$$

The variances of measurement noises are determined by characteristics of measurement sources. In this paper, the roll angle *φ* and pitch angle *θ* are calculated from the measured gravity in Equation (8) and the heading angle originate from polarization sensor in Equation (6). Therefore, the measurement noise variance can be represented by:

(30) $${\tilde{R}}_{k}=\left[\begin{array}{cccc} \sigma_{{}_{\phi }}^{2} & 0 & 0 & 0\\ 0 & \sigma_{{}_{\theta }}^{2} & 0 & 0\\ 0 & 0 & \sigma_{\psi }^{2} & 0\\ 0 & 0 & 0 & \sigma_{C}^{2} \end{array}\right],$$

where $\sigma_{\theta }^{2},\;\sigma_{\phi }^{2}$ are the variance of pitch and roll angles (*θ*, *φ*), $\sigma_{\psi }^{2}$ represents the heading angle variance, $\sigma_{C}^{2}$ represents the constraint variance in Equation (13) and in this equation $\sigma_{C}^{2}$ should not be selected as zero to avoid practical issues of the filter. Thus, the variance of roll and pitch angles $\sigma_{\theta }^{2},\;\sigma_{\phi }^{2}$ is defined [21] by:

(31) $$\left[\begin{array}{cc} \sigma_{\theta }^{2} & 0\\ 0 & \sigma_{\phi }^{2} \end{array}\right]=M_{\phi \theta }M_{xyz}R_{\theta \phi }M_{xyz}^{T}M_{\phi \theta }^{T}$$

where:

(32) $$M_{xyz}=\left[\begin{array}{llllllllllll} 1 & 0 & 0 & -1 & 0 & 0 & 0 & \omega_{z} & -\omega_{y} & 0 & -W & V\\ 0 & 1 & 0 & 0 & -1 & 0 & -\omega_{z} & 0 & \omega_{x} & W & 0 & -U\\ 0 & 0 & 1 & 0 & 0 & -1 & \omega_{y} & -\omega_{x} & 0 & -V & U & 0 \end{array}\right]$$

(33) $$M_{\phi \theta }=\left[\begin{array}{lll} 0 & \frac{g_{x}}{g_{y}^{2}+g_{z}^{2}} & -\frac{g_{y}}{g_{y}^{2}+g_{z}^{2}}\\ \frac{-g_{z}\cos\phi }{g_{x}^{2}\cos^{2}\phi +g_{z}^{2}} & \frac{g_{x}g_{z}^{2}\sin\phi }{\left(g_{y}^{2}+g_{z}^{2}\right)\left(g_{x}^{2}\cos^{2}\phi +g_{z}^{2}\right)} & \frac{\left(g_{y}^{2}+g_{z}^{2}\right)g_{z}\cos\phi -g_{x}g_{y}g_{z}\sin\phi }{\left(g_{y}^{2}+g_{z}^{2}\right)\left(g_{x}^{2}\cos^{2}\phi +g_{z}^{2}\right)} \end{array}\right]$$

(34) $$R_{\phi \theta }=diag\left[\begin{array}{cccccccccccc} \sigma_{ax}^{2} & \sigma_{ay}^{2} & \sigma_{az}^{2} & \sigma_{\dot{U}}^{2} & \sigma_{\dot{V}}^{2} & \sigma_{\dot{W}}^{2} & \sigma_{U}^{2} & \sigma_{V}^{2} & \sigma_{W}^{2} & \sigma_{\omega_{x}}^{2} & \sigma_{\omega_{y}}^{2} & \sigma_{\omega_{z}}^{2} \end{array}\right]$$

According to Equation (6), the heading angle *ψ* is connected with the pitch and roll angles, therefore the variance of heading angle $\sigma_{\psi }^{2}$ relates to them in the following way:

(35) $$\sigma_{\psi }^{2}=M_{\psi }E\left(\left[\begin{array}{c} \tilde{\alpha }\\ \tilde{\theta }\\ \tilde{\phi } \end{array}\right]{\left[\begin{array}{c} \tilde{\alpha }\\ \tilde{\theta }\\ \tilde{\phi } \end{array}\right]}^{T}\right)M_{\psi }^{T}=M_{\psi }R_{\psi }M_{\psi }^{T}$$

where:

(36) $$M_{\psi }=\frac{1}{\sqrt{1-b^{2}}}\left[\begin{array}{ccc} \frac{\partial b}{\partial \alpha } & \frac{\partial b}{\partial \theta } & \frac{\partial b}{\partial \phi } \end{array}\right]+\frac{1}{1+c^{2}}\left[\begin{array}{ccc} \frac{\partial c}{\partial \alpha } & \frac{\partial c}{\partial \theta } & \frac{\partial c}{\partial \phi } \end{array}\right]$$

where *b*, *c* are the parameters in the Equation (6),

And:

(37) $$R_{\psi }=diag\left[\begin{array}{ccc} \sigma_{\alpha }^{2} & \sigma_{\theta }^{2} & \sigma_{\phi }^{2} \end{array}\right]$$

where $\sigma_{\alpha }^{2}$, $\sigma_{\theta }^{2}$ and $\sigma_{\phi }^{2}$ are the variances of *α*, *θ* and *φ*, respectively.

## 5. Experimental Results and Discussion

### 5.1. Hardware Configuration and Scenario Description

**Figure 5:**
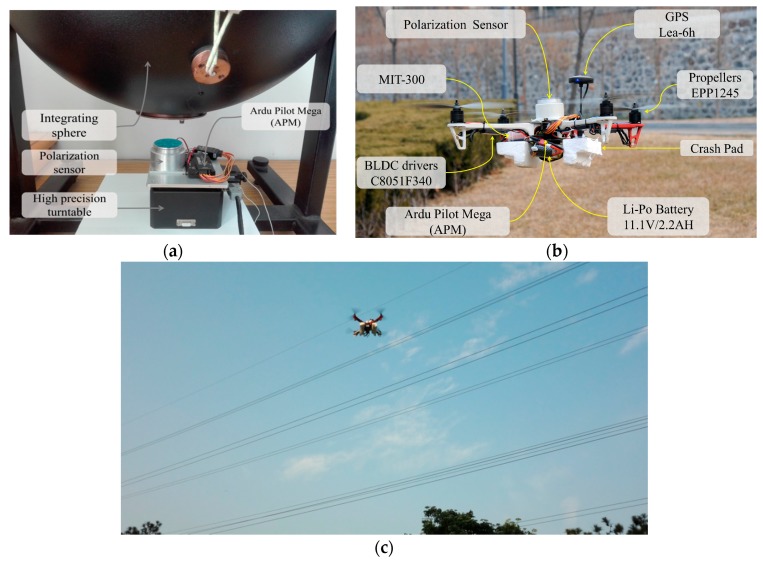
Experimental setup: (**a**) Indoor experimental setup; (**b**) Outdoor experimental setup; (**c**) The quad-rotor UAV flight near by the high voltage cables.

The test rig was composed of polarization sensor, GPS and open source flight controller Ardu Pilot Mega (APM). The APM [26] contained a Micro Inertial Measurement Unit (MIMU) mpu-6000 and a magnetometer HMC5883L. The specifications of all sensors are listed in Table 1. All the sensors data were collected and time-synchronized by the APM module. To evaluate the performance of the proposed algorithm, we conducted two experiments. The first experiment was carried out indoors, Figure 5a. The comparison of yaw angle was made between the APM output and polarization sensor output based on a high precision turntable. Furthermore, the magnetic field interference was added into the test section to test the reliability of polarization sensor working in the high magnetic field condition. The second experiment was executed outdoors in electromagnetic interference environment, Figure 5b,c. The novel attitude determination system and APM were mounted on a quad-rotor UAV. Using the commercial high-accuracy MEMS AHRS MTI-300, the computational results of two algorithms were compared.

### 5.2. Indoor Test

As presented in [27], the precision of polarization sensor can be limited to 0.2° by precision turntable calibration. However, few studies compared the existing AHRS with the polarization sensor indoor (or the system including the polarization sensor) to discuss the performance of these two systems working at disturbance of super magnetic field. This section focuses on that. The polarization sensor and APM module were fixed on the high precision turntable (accuracy of ±0.001°), which was placed under the integrating sphere, as shown in Figure 4. The all rigs on the turntable rotated around with the angular velocity of 2°/s and followed the turntable. The turntable is installed almost horizontally. The integrating sphere provided the homogeneous polarized light for the polarization sensor, which was used to emulate sun.

**Figure 6:**
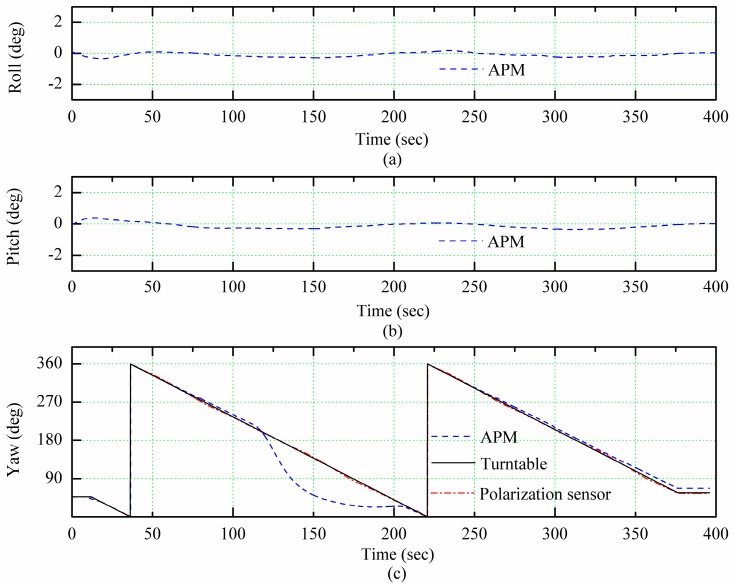
The comparison of attitude angles of polarization sensor and Ardu Pilot Mega (APM). (**a**) Roll, (**b**) Pitch, (**c**) Yaw.

In the test, the turntable made two circles and the test lasted for about 400 s. In the interval between 110 s and 160 s, a strong magnet was placed at the front of the turntable. To test the polarization sensor in the presence of magnetic disturbance, directly the output of the polarization sensor is given in Figure 6 without any processing with the EKF. It can be seen in Figure 6 that the heading angle output of APM result in great errors due to the magnet impact. However, at 160 s, although the magnet interference was eliminated, the heading angle of the APM output was not corrected immediately but had a delay of about 50 s. This is because the measurement at the previous moment is used as a predictive value in the iterative process of APM algorithm. Therefore, even though the interference was eliminated, the wrong measurement result at that moment still had some influence on the subsequent calculation accuracy. After a certain number of iterations, the more accurate measurement was achieved. Thus, although the magnetometer was combined with other sensors to obtain the heading angle, for instance in APM the heading angle was obtained by combination of magnetometer and gyroscope, the interference magnetic field still had a certain impact on measurement accuracy. However, the output of the polarization sensor was not affected by magnetic field interference and had a certain robustness. Since the polarization sensor was kept in a horizontal state all the time, its output without correction could also maintain high precision. Furthermore, attitude information of the APM was not affected by a strong magnetic field, because the data represent the fusion of accelerometers and gyroscopes data.

Between 220 s and 400 s, there was no magnetic field interference. The root mean square error of the output angle of APM was 1.57° and the root mean square error of the output angle of polarization sensor was 0.83°. Comparing the performance of two rigs in the entire test, the output of polarization sensor had higher precision and stronger anti-magnetic interference ability.

### 5.3. Outdoor Test

**Figure 7:**
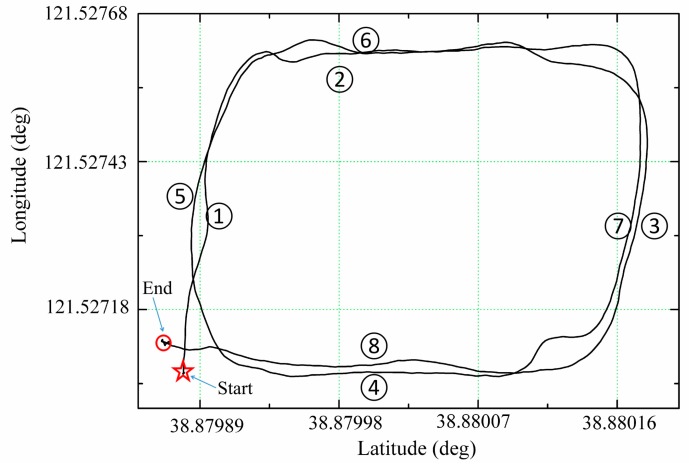
The flight path of flying quad-rotor UAV.

In the flight test scenario, the attitude precision of APM and proposed system was compared and both systems were tested on a quad-rotor UAV. In the comparison, the flight data from high accurate MEMS AHRS (MTI-300) were employed as a reference. The flying quad-rotor UAV carried APM, polarization sensor and GPS receiver as shown in Figure 5b and the flight lasted for about 155 s. The test was performed between 3:30 P.M. and 4:30 P.M. to avoid direct sunlight effect on the polarization sensor. The solar altitude angle was about 29° and azimuth angle was about 93°. The flight was performed on a square in Dalian University of Technology (38.879° N, 121.527° E) on 21 December 2017. Above the square there were few high voltage cables which produced a certain electromagnetic field. In order to test and verify the effect of electromagnetic field on magnetometer, the aircraft got close to the high voltage cables two times. The location data recorded by GPS are shown in Figure 7. As shown in Figure 7, in flight segments 2 and 6, the UAV was near to the high voltage cables.

**Figure 8:**
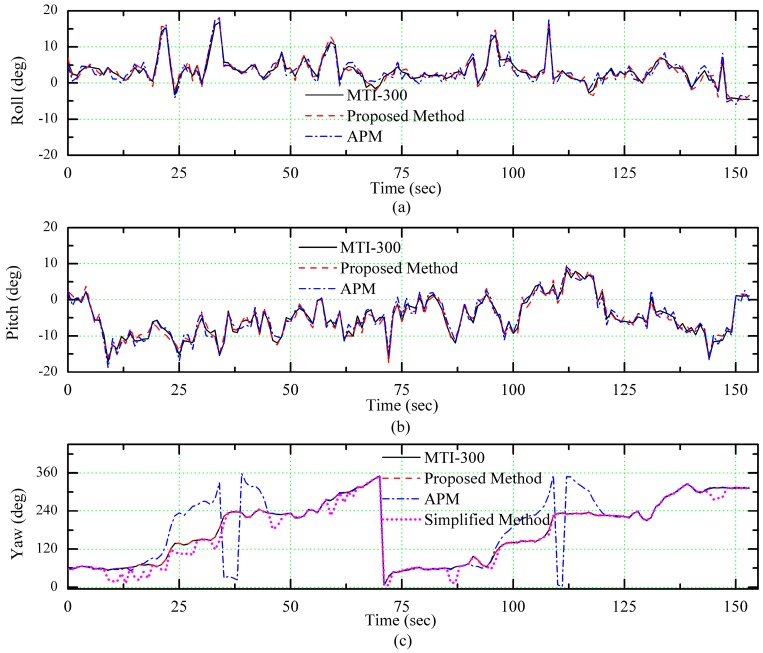
The estimated attitude over time. (**a**) Roll, (**b**) Pitch, (**c**) Yaw.

In Figure 7, we can see that there were many turns in the flight path and these turning behaviors made the vehicle inclined, so the polarization sensor on the vehicle tilted accordingly. Figure 8 shows the attitude data during the flight, which were obtained by high accurate AHRS (MTI-300), the APM, the simplified method and the proposed algorithm. The simplified method is using the simple model for calculating the heading angle using polarization sensor measurements and neglecting the sensor tilt.

As Figure 8 shows, the algorithm proposed in this paper can effectively calculate the dynamic attitude data of the vehicle and there is a significant deviation in the heading angle of the APM output when the aircraft approaches the high voltage cables. The significant deviation appeared in APM output from 14 s to 25 s and from 90 s to 121 s, which corresponded to segments 2 and 6 in Figure 7. Moreover, we can see that when the vehicle tilted and its pitch and roll angles changed for more than 10°, there were some large deviations in heading angle obtained by the simplified algorithm but the heading angle calculated by the proposed algorithm was equal to the actual heading angle of the UAV. 

**Figure 9:**
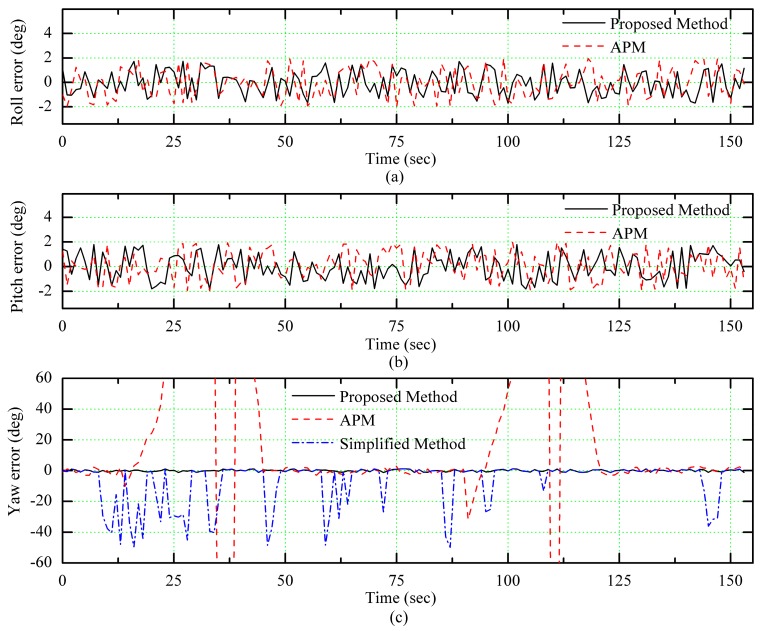
The attitude estimation error. (**a**) Roll error, (**b**) Pitch error, (**c**) Yaw error.

In order to observe the measurement results of different sensors directly, the attitude errors were calculated with the output of MTI-300 as a reference. In Figure 9, we can see that the precision of pitch and roll angles measured with the proposed algorithm corresponds to the precision of APM and the precision of heading angle measured with the proposed algorithm is improved significantly in comparison between those of APM and the simplified algorithm. 

The mean square error of all measurements is presented in Table 2. It can be seen from the table that due to the influence of the tilt of the aircraft at some stages, the mean square error of yaw calculated by the simplified algorithm is quite large. Consequently, the attitude obtained with the proposed algorithm is more precise than others.

## 6. Conclusions

In this paper, a novel attitude determination system with a self-made polarization sensor is proposed. In the proposed system, the magnetometer of the traditional attitude reference system is replaced with the polarization sensor. The GPS data are introduced to correct the error in attitude measurement of the motion vector. The output data of all sensors in the system are fused by EKF. The proposed system was verified by two experiments, the indoor test and outdoor test. The indoor test results showed that polarization sensor is not affected by magnetic field, which enables proposed system to work properly in magnetic interference environments. The outdoor test results showed that proposed system has higher measurement accuracy than commonly used attitude determination system and can provide the precise parameters for UAV flight control. 

In the future, we will improve the proposed system. The gyro bias terms could be estimated to correct measured angular velocities at each recursive step of EKF, based on algorithms proposed by Markley. In addition, a higher performance microprocessor should be adopted to deal with a large amount of computation induced by a high-dimensional state variable. Moreover, the roll and pitch angles estimations from the filter could be used to correct heading angle instead of the accelerometer measurements, with the aim of achieving a more accurate heading angle. In addition, the quaternion could be employed instead of Euler Angle as a measurement value to avoid a heavy Jacobian matrix calculation and the corresponding measurement noise model should be also derived. All of the above methods may contribute to improve the efficiency and accuracy of the system

## Figures and Tables

**Table 1:** The accuracy specifications of sensors.

Sensors	Product Model	Specifications	Sampling Rates (Hz)
AHRS	MTI-300	Roll dynamic value: 0.3°	100
		Pitch Dynamic value: 0.3°	
		Yaw Dynamic value: 1.0°	
MIMU	MPU6000	Gyro Sensitivity: 131 LSB/(°/s) Full scale range: ±250°/s	20
		Accelerometer Sensitivity: 4096 LSB/g Full scale range: ±8 g	
Magnetometer	HMC5883	Typical Noise Floor: 2 milligauss	10
Polarization sensor	Self-development	Accuracy (indoor): 0.2° Accuracy (outdoor): <2°	10

**Table 2:** Flight test error statistics.

Methods	Roll (RMSE)	Pitch (RMSE)	Yaw (RMSE)
APM	1.93°	1.89°	3.01°
Proposed Method	1.87°	1.85°	1.25°
Simplified Method	Null	Null	23.10°
